# CREB1 is affected by the microRNAs miR-22-3p, miR-26a-5p, miR-27a-3p, and miR-221-3p and correlates with adverse clinicopathological features in renal cell carcinoma

**DOI:** 10.1038/s41598-020-63403-y

**Published:** 2020-04-16

**Authors:** Michael Friedrich, Nadine Heimer, Christine Stoehr, André Steven, Sven Wach, Helge Taubert, Arndt Hartmann, Barbara Seliger

**Affiliations:** 1 Institute for Medical Immunology, Martin Luther University Halle-Wittenberg, 06112 Halle (Saale), Germany; 2 Institute of Pathology, University Erlangen-Nürnberg, 91054 Erlangen, Germany; 3 Institute of Urology, University Erlangen-Nürnberg, 91054 Erlangen, Germany

**Keywords:** Immunogenetics, Non-coding RNAs

## Abstract

The transcription factor *cAMP response element-binding protein* (CREB1) has been shown to be involved in diverse biological pathways including the regulation of cell proliferation, apoptosis, cell cycle progression, and metastasis. In this context, aberrant expression of CREB1 and the functional consequences are well investigated in a number of hematopoietic and solid tumors. However, CREB1 expression and underlying control mechanisms are only poorly analyzed in renal cell carcinoma (RCC). The present study confirmed a deregulation of CREB1 protein in the clear cell type of RCC (ccRCC) and analysis of in-house ccRCC cell lines suggested a post-transcriptional control. The combination of miRNA enrichment assay, *in silico* analysis and molecular biological approaches revealed four novel CREB1-regulating miRNAs, namely miR-22-3p, miR-26a-5p, miR-27a-3p, and miR-221-3p. Categorizing RCC samples as CREB1 negative or positive, respectively, the expression of these miRNAs was found to be inversely correlated with CREB1 protein levels. Analyzing 453 consecutive RCC tumors by immunohistochemistry, weakly negative, but significant correlations of CREB1 with tumor stage and grade, vascular invasion (V1) and lymphovascular invasion (L1) were found. In this respect, ccRCC might differ from other solid tumors like esophageal squamous-cell carcinoma or glioma.

## Introduction

Renal cell carcinoma (RCC) is a kidney cancer originating in the lining of proximal renal tubular epithelial cells and can be histologically subtyped into clear cell type (ccRCC, 75%), papillary (pRCC, 13–15%), chromophobe type (chRCC, 5%), and several rare subtypes^1,2^. Globally, RCC is listed as the sixth most frequently diagnosed form of cancer in men and the tenth in women, with highest rates described in North America and the Czech Republic^3,4^. Noteworthy, RCC incidence was shown to be increasing and frequently accompanied by several risk factors including smoking, hypertension, obesity, chronic analgesic use, and diabetes^5,6^. Early treatment options for RCC are quite limited and usually involve partial or complete resection of the affected kidney(s)^7^ since this type of tumor is considerably resistant to chemotherapy and radiotherapy^8^. However, immunotherapy as well as biologic or targeted therapy has shown to be an efficient option for RCC treatment^9^. Thus, it is important to characterize molecular targets responsible for the formation and development of this disease. Probably best analyzed is the alteration of the tumor suppressive *Von Hippel-Lindau* (VHL) gene that has a crucial value in the origin and development of ccRCC and can be found to be affected in up to 90% of all ccRCC cases^10^. Situated in a complex pathway, VHL is ultimately responsible for the regulation of the transcription factors hypoxia-inducible factor 1 alpha and 2 alpha (HIF1A, HIF2A)^11,12^. The deregulation of HIF1A and HIF2A results in the up-regulation of various growth factors like vascular endothelial growth factor (VEGF), platelet-derived growth factor beta (PDGFB), and transforming growth factor alpha (TGFA) responsible for the development of the tumor. These growth factors are currently targeted by specific inhibitors (e.g. sunitinib and pazopanib) as first-line therapy option for metastatic ccRCC^13^.

The transcription factor *cAMP response element-binding protein* 1 (CREB1) might possibly be another interesting target for this kind of therapy. The basic leucine zipper motif-containing CREB1^14^ possesses responsive elements (CRE sites) in over 4,000 gene promoters^15^. This could be the reason for the broad range of biological pathways regulated by CREB1 including differentiation and cell growth^16^. In this context, CREB1 harbors a high oncogenic potential and is capable to be part of the malignant transformation converting normal cells into tumor cells^17^. In hematopoietic (e.g. acute myeloid leukemia) and some solid tumors (e.g. melanoma, glioblastoma) CREB1 was found to be overexpressed resulting in increased cell proliferation, suppressed apoptosis, and enhanced angiogenesis and differentiation^17,18^.

In RCC, only a limited number of studies evaluated the impact of CREB1 and tumor development and progression. For instance, Zhuang and co-authors showed that CREB1 regulates *spindle and kinetochore-associated protein 2* (SKA2) on transcriptional level. The overexpression of SKA2 by up-regulated CREB1 promotes RCC cell proliferation *in vitro* and *in vivo*^19^. In 2017, Wang and co-worker found CREB1 to be a regulator of epithelial to mesenchymal transition (EMT) in RCC probably by controlling the expression of matrix metallopeptidase (MMP) 2/9 and further proteins involved in EMT^20^. In a follow-up study, MMP 2/9 were found to be decreased in sorafenib-treated RCC cells characterized by inhibited cell migration and invasion while the artificial overexpression of CREB1 reversed sorafenib-induced effects in these cells^21^.

However, CREB1 expression and the underlying control mechanisms are only poorly analyzed in RCC. In this study, we aimed to analyze the CREB1 status in ccRCC samples and investigated putative post-transcriptional regulation mechanisms responsible for the deregulation of CREB1 in the tumors. Furthermore, correlation studies were performed, and the question addressed whether CREB1 and CREB1-regulating miRNA expression have clinical relevance in ccRCC patients.

## Results

### CREB1 protein is aberrantly expressed in ccRCC

While overexpression of CREB1 is well described in a number of cancer types including solid (e.g. melanoma, glioblastoma) and hematopoietic tumors (e.g. acute myeloid leukemia)^17,18^, CREB1 expression and the underlying control mechanisms are only poorly analyzed in RCC. Therefore, CREB1 expression was investigated in ccRCC as this is the most common histological type of this disease.

**Figure 1:**
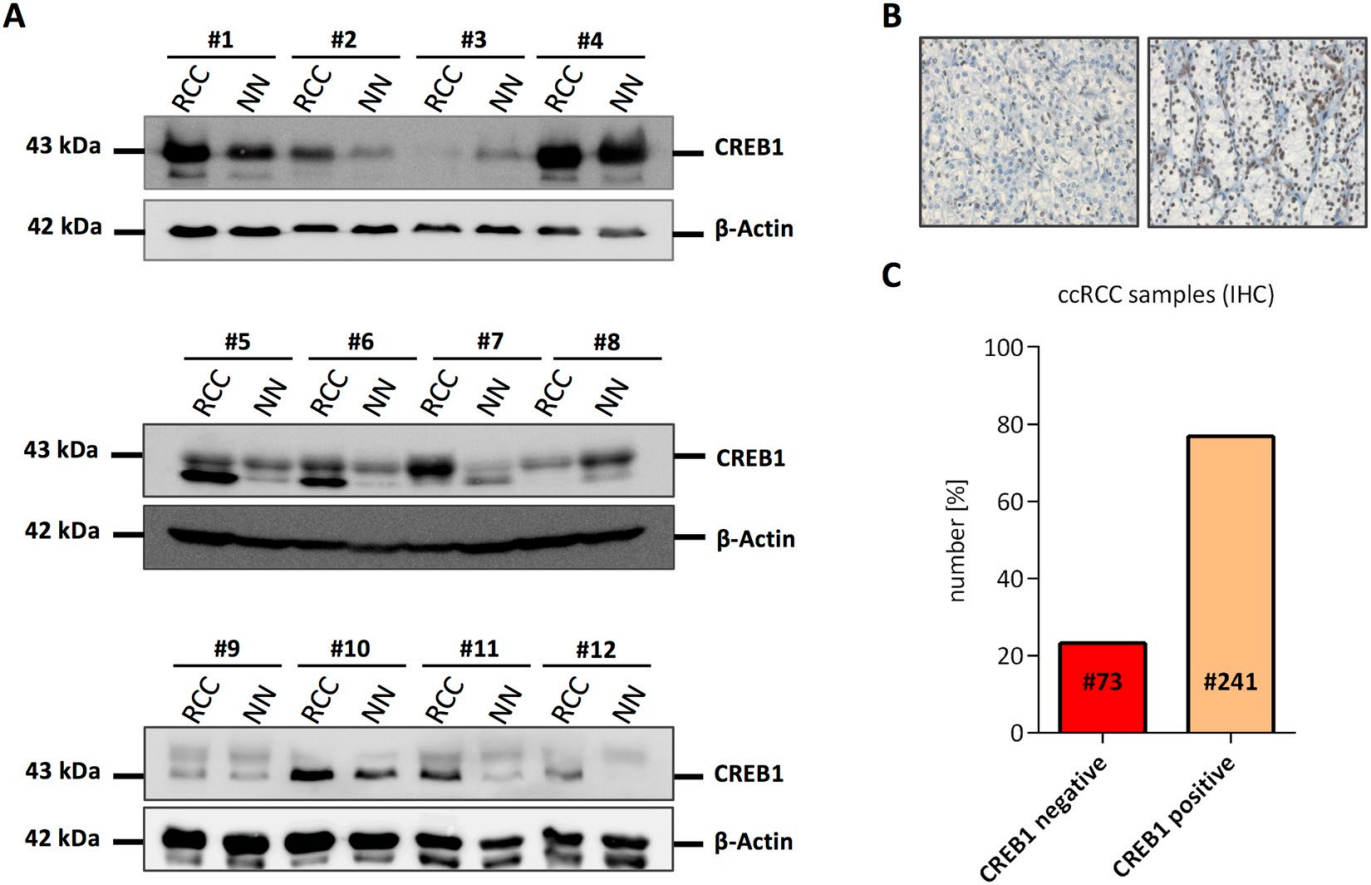
CREB1 protein expression in ccRCC tumor samples. (**A**) Western blot-based detection of CREB1 in 12 pairs of ccRCC tumor samples (RCC) and matched adjacent normal tissue (NN). Reduced CREB1 protein levels were observed in sample #3 and #8. The same blot was re-probed for β-Actin which served as loading control. Uncropped blots are shown in Supplementary Figure 1. Relative CREB1 quantification is shown in Supplementary Figure 2. (**B**) Two representative immunohistochemical stainings of a CREB1 negative (left) and a CREB1 positive (right) RCC lesion from a TMA consisting of 453 consecutive tumors are shown. (**C**) Results of panCREB1 immuno-histochemistry. Only data on clear cell RCC are shown. While most ccRCC were CREB1 positive, more than 20% were classified negative.

First, CREB1 protein expression was analyzed in 12 pairs of ccRCC tissues and matched adjacent non-tumor tissues by Western blot, which demonstrated an aberrant expression. Confirming recent studies^19,22^, CREB1 protein was frequently up-regulated (9/12 pairs, 75%) in the tumor tissue compared to matched non-tumor tissue. Noteworthy, in two out of twelve pairs (~17%) CREB1 was down-regulated in the tumor suggesting a different regulation in this CREB1 low subtype (Fig. 1A and Supplementary Figure 2). To further validate this pattern, 314 ccRCC FFPE samples located on a consecutive RCC TMA were analyzed by IHC using an α-panCREB1 specific mAb. Representative stainings of CREB1 ccRCCs expressing low and high levels of CREB1 protein are shown in Fig. 1B. Although highly expressed in kidney^23^, ~23% of all samples were CREB1-negative confirming the existence of a CREB1 low subtype in ccRCC (Fig. 1C).

### ccRCC cell line MZ2733RC as a model to identify CREB1-regulating miRNAs

**Figure 2:**
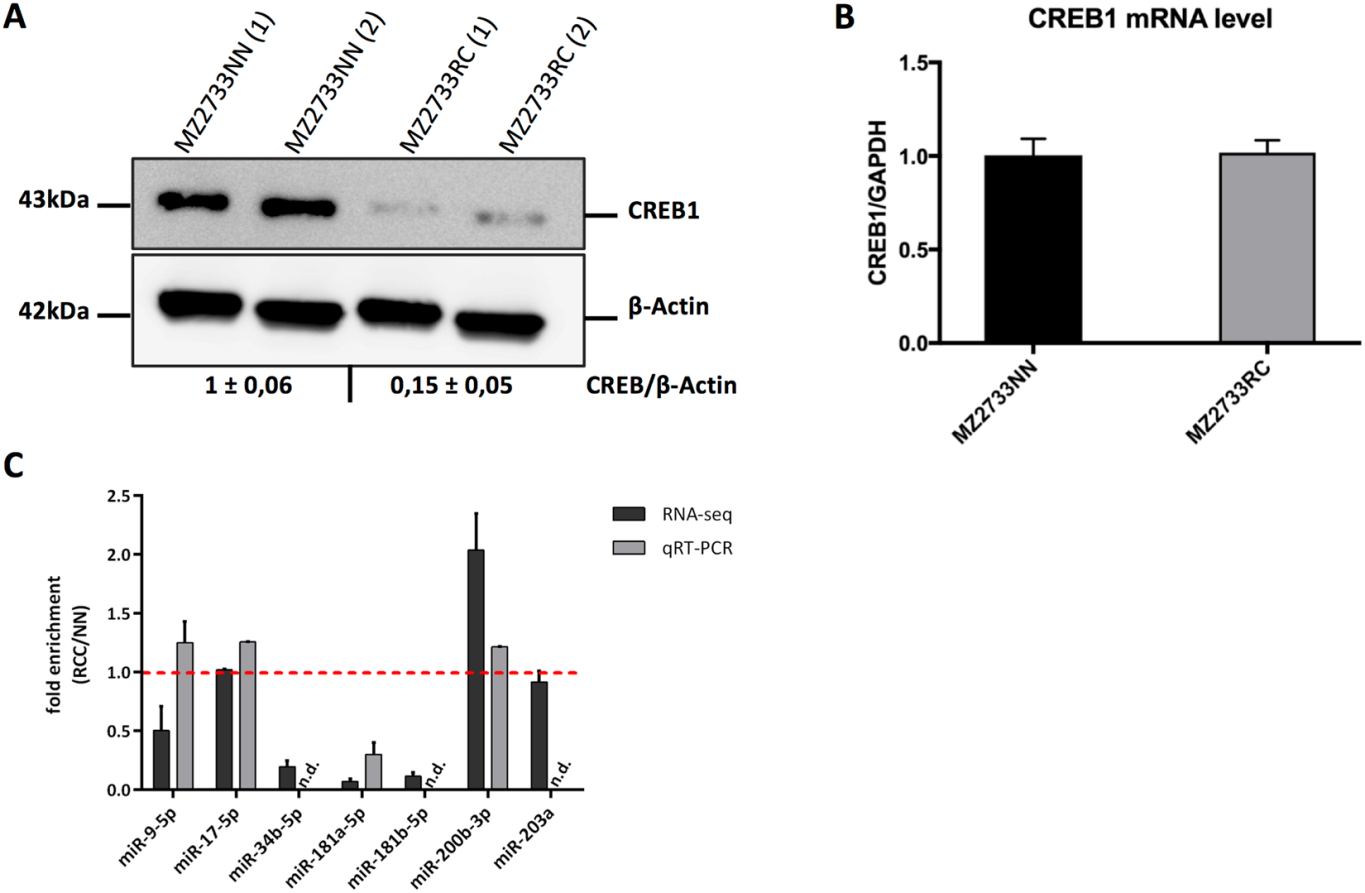
ccRCC cell line MZ2733RC as a model for the identification of unknown CREB1-specific post-transcriptional regulators. (**A**) Western blot-based detection and densitometric quantification of CREB1 protein in the ccRCC cell line MZ2733RC and the corresponding normal tissue cell line MZ2733NN. Number in parentheses indicates a technical replicate. Same blot was re-probed for β-Actin which served as loading control. Uncropped blot is shown in Supplementary Figure 3. (**B**) CREB1 mRNA levels analyzed by qRT-PCR in the indicated cell lines. GAPDH was used for normalization. (**C**) Expression levels of validated CREB1-specific miRNAs given as fold enrichment for MZ2733RC (RCC) cell line relative to MZ2733NN (NN) cell line analyzed by RNA-sequencing and qRT-PCR. Values above 1 (red line) indicate miRNAs enriched in the tumor cell line. n.d. not detectable.

The ccRCC cell line MZ2733RC and the corresponding normal tissue cell line MZ2733NN showed a divergence in CREB1 protein expression (Fig. 2A). However, CREB1 mRNA levels were highly comparable in both cell lines pointing to an extensive post-transcriptional regulation of CREB1 in the tumor cell line MZ2733RC (Fig. 2B). Thus, MZ2733RC was selected for further analysis focusing on the identification of miRNAs as post-transcriptional regulators of CREB1. To determine whether the known CREB1-specific miRNAs are up-regulated in MZ2733RC and therefore responsible for low CREB1 protein levels, the expression of the seven best validated CREB1-specific miRNAs, namely miR-9, -17, -34, -181a, -181b, -200b, and miR-203a were analyzed in MZ2733RC and MZ2733NN by small RNA-seq and qRT-PCR (Fig. 2C). While three analyzed miRNAs are strongly down-regulated in the RCC cell line (miR-34, -181a, -181b), none of them is substantially up-regulated and could be the cause for the low CREB1 protein abundance. As a consequence, MZ2733RC cell line may provide an excellent starting point for the identification of new CREB1-regulating miRNAs.

### miRNA trapping by RNA *in vitro* affinity purification

**Figure 3:**
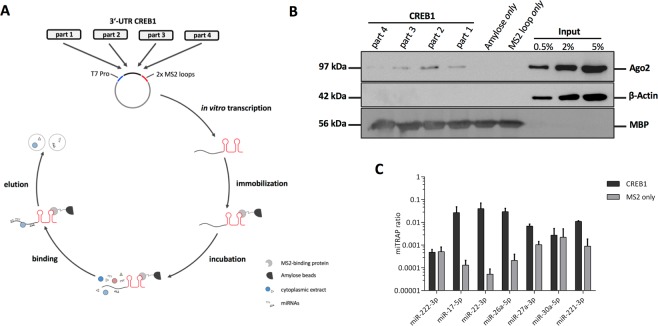
miTRAP analyzing 3′-UTR of CREB1. (**A**) Scheme illustrating the miTRAP method using 3′-UTR of CREB1. (**B**) Western blot-based detection of indicated proteins isolated from the miTRAP input sample (MZ2733RC) or co-precipitated with the used resin (amylose only), 2x MS2 loop RNA (MS2 loop only) or the different parts of the 3′-UTR of CREB1, respectively. Same blot was re-probed for β-Actin serving as negative control to exclude unspecific binding to the different RNAs. Detection of maltose binding protein (MBP) on the same blot ensures equal loading of the resin. Uncropped blot is shown in Supplementary Figure 4. (**C**) Co-purification of candidate miRNAs after the miTRAP procedure using CREB1 3′-UTR or MS2 loop control analyzed by qRT-PCR. Values are normalized to the input expression. miR-222–3p served as negative control (part 1) while miR-17–5p (part 2) was used as positive control.

For the identification of putative CREB1-specific miRNAs a combination of *in silico* analysis and RNA *in vitro* affinity purification (miTRAP) was performed^24^. First, the 8,944 nt long 3′-UTR of CREB1 was separated in four overlapping parts of ~2,250 nt in size and inserted each part upstream of two MS2 repeats enabling the immobilization of the *in vitro* transcript by an MS loop antibody (Fig. 3A). Subsequently, *in vitro* transcripts were used for RNA affinity purification and co-purified proteins were analyzed by Western blot. An increased incidence of the RISC core component AGO2 at the 3′-UTR of CREB1 indirectly pointed to the binding of miRNAs (Fig. 3B). Since part 1–3 showed enriched levels of AGO2, our further *in silico* analysis was focused on these 3′-UTR sections. Using different miRNA prediction algorithms including Targetscan^25^, RNAhybrid^26^, miRanda^27^, and miRWalk2.0^28^ miR-22–3p, miR-26a-5p, miR-27a-3p, miR-30a-5p, and miR-221-3p were identified as novel putative CREB1-specific miRNAs (Table 1).

**Table 1:** *In silico* identification of putative CREB1-specific miRNAs.

miRNA	binding prediction			
	Targetscan	RNAhybrid	miRanda	miRwalk
hsa-miR-22-3p	no	yes	yes	yes
hsa-miR-26a-5p	yes	yes	yes	yes
hsa-miR-27a-3p	yes	yes	yes	yes
hsa-miR-30a-5p	yes	yes	yes	yes
hsa-miR-221-3p	yes	yes	yes	yes

To investigate the potential of the identified miRNAs to bind to the 3′-UTR of CREB1, miTRAP was performed using the 3′-UTR MS2 loop fusion constructs. For this purpose, the miRNA abundance in the miTRAP eluate fraction normalized to the input (miTRAP ratio) was determined by qRT-PCR. For better classification, the already published CREB1-regulating miR-17-5p served as positive control, while miR-222-3p served as negative control. Except miR-30a-5p, an accumulation of the *in silico* predicted miRNAs in the CREB1 eluate compared to the MS2 control was confirmed (Fig. 3C). Therefore, miR-30a-5p was excluded from further experiments and the remaining four miRNA candidates were used for validation and functional analysis.

### Validation of CREB1-regulating miRNA candidates

**Figure 4:**
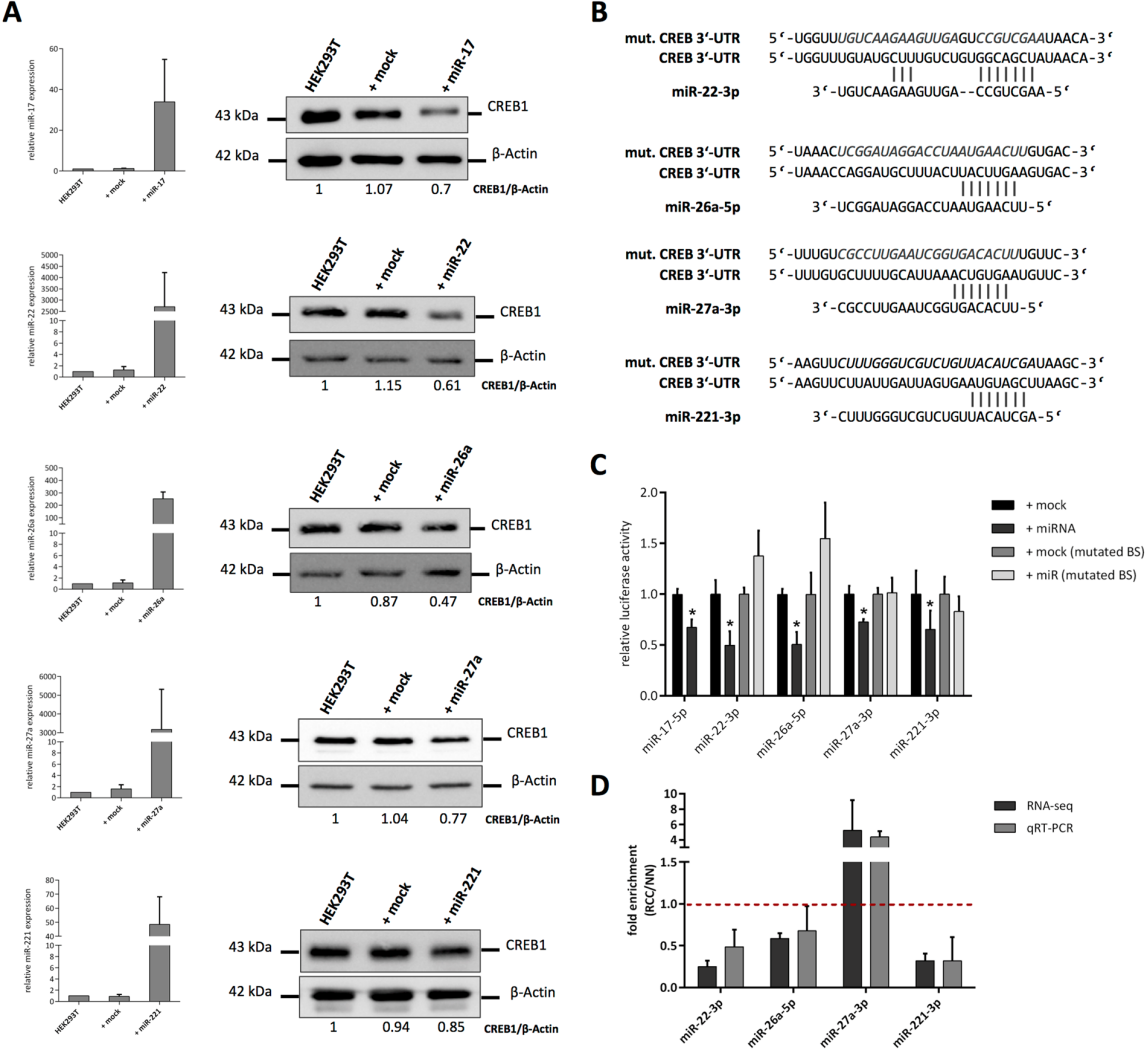
Validation of CREB1-regulating miRNA candidates. (**A**) Effect of miRNA mimic or mock transfection on respective miRNA expression in HEK293T cells (left). Western blot-based detection and densitometric quantification of CREB1 after the miRNA mimic or mock transfection in HEK293T cells was performed as described in Materials and Methods. Same blot was re-probed for β-Actin which served as loading control (right). Uncropped blots are shown in Supplementary Figure 5. (**B**) Illustration of the predicted candidate miRNA binding sites in the 3′-UTR of CREB1 and the mutated versions later used for luc reporter gene assay. (**C**) Relative luc activity measured after transfection of indicated miRNA mimics or mock control using wt CREB1 3′-UTR or binding site (BS) mutated versions (n = 4, student t-test, *p < 0.05). (**D**) Analysis of indicated candidate miRNA expression levels given as fold enrichment for MZ2733RC (RCC) cell line relative to MZ2733NN (NN) cell line analyzed by RNA-sequencing and qRT-PCR. Values above 1 (red dotted line) indicate miRNAs enriched in the tumor cell line.

To verify the effect of the miRNA candidates on the expression of CREB1, the embryonal kidney cell line HEK293T was transfected with the respective miRNA mimics using miR-17-5p as a positive control. All miRNA mimic transfections resulted in significant up-regulated levels of the respective mature miRNA expression. Furthermore, overexpression of all selected miRNA candidates led to the down-regulation of CREB1 protein (Fig. 4A). Interestingly, the level of protein down-regulation correlated with the enrichment of the miRNAs in the miTRAP assay, which were highest for miR-22-3p and miR-26a-5p and the lowest for miR-27a-3p and miR-221-3p.

In order to exclude an indirect/secondary effect responsible for the down-regulation of CREB1 protein after overexpression of the respective miRNA as well as to determine the exact miRNA binding site in the 3′-UTR of CREB1, luciferase (luc) reporter gene assays were performed using the wild type (wt) 3′-UTR or a mutated version of the miRNA binding site (Fig. 4B,C). A downregulation of the relative luc activity after transfection of the miRNA mimics was found for all candidates when compared to the mock control. The mutation of the expected miRNA binding site in the 3′-UTR of CREB1 abolished this effect indicating a direct interaction of miR-22-3p, miR-26a-5p, miR-27a-3p, and miR-221-3p with the predicted sequence.

Next, it was analyzed whether the newly validated CREB1-specific miRNAs were responsible for low CREB1 levels in the model cell line MZ2733RC. The analysis of miRNA expression by RNA-seq and qRT-PCR revealed a strong up-regulation (~5-fold) of miR-27a-3p in the tumor cell line relative to MZ2733NN, while the remaining miRNAs showed a lower expression (Fig. 4D). Altogether, these data suggest that miR-27a-3p is at least partially involved in the post-transcriptional regulation of CREB1 in the tumor cell line MZ2733RC.

### Correlation of CREB1 and newly identified miRNAs

**Figure 5:**
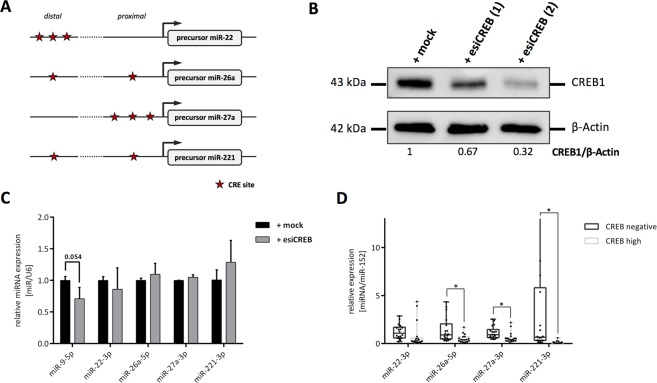
Correlation of CREB1 and newly identified CREB1-specific miRNAs. (**A**) *In silico* prediction of miRNA promoter and identification of CREB1 responsive elements (CRE sites, red asterisk) within these sequences. Proximal promoter reflects 1,000 bp. (**B**) Western blot-based detection of CREB1 and densitometric quantification after transfection of esiRNAs specific to CREB1 in MZ2862RC cell line. The number in parentheses indicates a technical replicate. Same blot was re-probed for β-Actin which served as loading control. Uncropped blot is shown in Supplementary Figure 6. (**C**) Effect of esiRNA mediated CREB1 knock-down on the expression level of indicated miRNAs. miR-9-5p was used as positive control (n = 3, student t-test). (**D**) Expression of the indicated miRNAs was determined in 18 CREB1 negative and 18 CREB1 high ccRCC lesions by qRT-PCR and results are visualized as Box-Whisker-Plots (Holm-Sidak method, miR-22: p = 0.269; *p  < 0.05).

Recently, Tan and co-workers were able to show that the transcription factor CREB1 is able to control the expression of the CREB1-regulating miR-9 and thereby forms a negative feedback loop critical for determination of “go or grow” in glioma^29^. Based on this, it was investigated whether CREB1 could modulate the expression of the identified miRNAs. As a first step, we used the tool miRStart^30^ to identify human microRNA transcription start sites and promoter sequences. Subsequently, PROMO database^31^ provided the possibility to predict putative CREB1 binding sites (CRE sites) in the respective miRNA promoter (Fig. 5A). We observed CRE sites in all CREB1-regulating miRNA and, thus, used endoribonuclease-prepared siRNAs (esiRNA, Sigma-Aldrich) to target CREB1 mRNA. CREB1 protein was downregulated in the ccRCC cell line MZ2862RC to approximately 32–65% (Fig. 5B). However, a deregulation of miR-22-3p, miR-26a-5p, miR-27a-3p, and miR-221-3p was not detectable. Only miR-9-5p was found to be reduced after CREB1 knock-down (Fig. 5C). Next, a putative correlation of CREB1 and the newly identified CREB1-specific miRNAs was determined *in situ*. The expression of the miRNAs was measured in 18 selected CREB1 negative and 18 CREB1 high ccRCC lesions located on the consecutive RCC TMA by qRT-PCR (Fig. 5D). Expression levels were significantly reduced for miR-26a, miR-27a-3p, and miR-221-3p, while miR-22-3p showed a non-significant tendency for lower RNA quantity in the CREB1 high samples.

### Correlation of CREB1 and clinical parameter in RCC

To address whether the panCREB1 expression in RCC impacts basic clinical parameters of different RCC subtypes, panCREB1 immunohistochemistry was performed on a consecutive RCC TMA. Due to tissue loss and lack of tumor cells, 400 of 453 RCC tumors could be evaluated for panCREB1 staining. 72.8% (291/400) of all RCC samples enclosed were positive for panCREB1 when using the binary classification system. No significant associations were found to patient characteristics, i.e. sex (p = 0.081) or age at diagnosis (p = 0.709).

PanCREB1 positivity differed significantly among tumor characteristics. There was a differential expression among the tested subtypes (p = 0.01). While ccRCC and pRCC displayed positivity in 62.8% (pRCC, 27/43) to 76.8% of cases (ccRCC, 241/314), chRCC were positive in only 44.4% of cases (12/27). There also was an association to WHO grade (2004, p < 0.001) and pT stage (p = 0.002). The highest amount of positive cases was found in grade 1 tumors (47/49, 95.9%), the lowest in grade 3 tumors (52/93, 55.9%). Similarly, pT1 was the tumor stage displaying the highest amount of positive cases (207/260). However, data on tumor stage should be interpreted with care, as 300 of 398 cases group to stage pT1 and pT2 and only 96 and 2 cases to pT3 and pT4, respectively.

**Figure 6:**
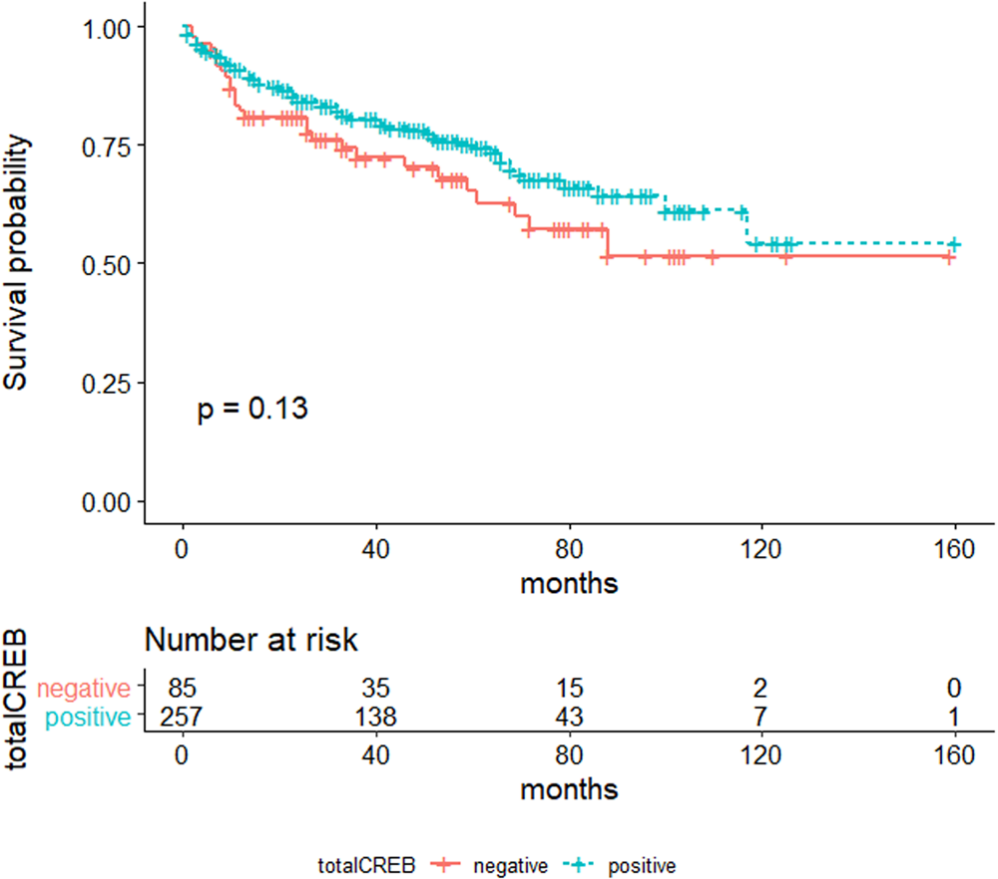
Cumulative Survival. Unadjusted overall survival of RCC patients with regard to tumoral panCREB1 expression. The panCREB1 negative group is shown in red, the panCREB1 positive group in blue. Number of events: panCREB1-negative: n = 28, panCREB-positive: n = 64. Censored cases: panCREB1-negative: n = 57, panCREB-positive: n = 193.

In addition, Spearman Rho correlations were performed on the ungrouped panCREB1 scores to test for the strength of correlations. As shown in Table 2, no correlation of CREB1 and RCC patients’ sex or age was observed, which is in line with the findings on binary staining categorization. Very weak to weak negative, but significant correlations were observed for CREB1 and T-stage, TNML, TNMV and WHO grade (2014) (Table 2). In accordance with these weak negative associations, the non-adjusted overall survival of patients with panCREB1 positive or panCREB1 negative tumors did not significantly differ (p = 0.134, n = 342) as shown in Fig. 6. In addition, no correlations of patient’s overall survival to CREB-expression was found, when adjusting for covariates. (Supplementary Table 1).

**Table 2:** Correlation of CREB1 expression and clinical characteristics.

		panCREB1
sex	correlation coefficient	−0.095
	p-value	0.056
	N	405
age at diagnosis	correlation coefficient	−0.037
	p-value	0.488
	N	346
pT stage	correlation coefficient	−0.300
	p-value	<0.001
	N	399
TNM L	correlation coefficient	−0.140
	p-value	0.032
	N	236
TNM V	correlation coefficient	−0.202
	p-value	<0.001
	N	250
WHO grade 2014	correlation coefficient	−0.299
	p-value	<0.001
	N	407

## Discussion

In line with a previous publication^19^, we found CREB1 to be frequently up-regulated in ccRCC tumors compared to corresponding adjacent normal tissue. This confirms the oncogenic potential of CREB1 as high protein levels were observed in a number of other solid tumors including breast cancer, non-small-cell lung cancer, diffuse malignant mesothelioma and melanoma^32–35^. In these types of cancer deregulation of CREB1 was shown to be associated with enhanced proliferation, reduced apoptosis and increased angiogenesis^17^. First reports for RCC demonstrated very similar observations^19,20^. In addition, our analysis showed a significant number of RCC samples with reduced CREB1 protein levels. If decreased CREB1 expression is also here involved in the malignant transformation of kidney cells need to be examined in further studies.

However, underlying mechanisms responsible for aberrant CREB1 expression in general are only poorly understood. Evaluating *The Cancer Genome Atlas* (TCGA) data using the cBioPortal database^36,37^, showed only low numbers (with the exception of neuroendocrine prostate cancer: 12%) of genetic alterations for CREB1 (e.g. gene amplification) ranging from 1.3% in ccRCC to 5% in ovarian serous cystadenocarcinoma. Analysis of the in-house established ccRCC cell line pair MZ2733RC/NN led to the conclusion that post-transcriptional gene regulation may be an important mechanism to control CREB1 expression. This is further supported by the fact that CREB1 3′-UTR with ~8,900 nt is well above the human average 3′-UTR size (~1,200 nt)^38^ suggesting an extensive post-transcriptional regulation via this region. Using the combination of miTRAP, *in silico* analysis and molecular biological approaches, we were able to identify four novel CREB1-regulating miRNAs, namely miR-22-3p, miR-26a-5p, miR-27a-3p, and miR-221-3p. These new miRNAs complete a list of already known CREB1-specific miRNAs including miR-9, -17-92 cluster, -27b, -34b, -181a/b, -182, -200b, -203, and miR-433^39^. However, in contrast to miR-34b, -181b and miR-203, miRNAs identified in the present study are characterized by a proper expression in RCC causal to regulate gene expression (see also Fig. 2C). Noteworthy, *in vitro* and *in cellulo* experiments point to miR-22-3p and miR-26a-5p as more effective in deregulating CREB1 protein expression. In this context, miR-22-3p may benefit from a more extensive binding of the non-seed region (3′-part of the miRNA) to CREB1 mRNA (see also Fig. 4B). Expression analysis in CREB1 negative and CREB1 high RCC samples revealed a significant inverse correlation of CREB1 and miR-26a-5p, miR-27a-3p, and miR-221-3p expression. Therefore, the miRNAs miR-26a-5p, miR-27a-3p, and miR-221-3p may provide a tool for diagnostic purposes identifying CREB1 negative/low and CREB1 high tumors and, thus, potentially important CREB1-regulated targets, such as members of the cyclin family, VEGF, and MMP-2^40–43^. Moreover, an implementation of miRNAs (or antogomiRs) has been suggested as novel therapeutic option^44^ and the here presented CREB1-specific miRNAs may be of interest for those approaches.

Finally, using a TMA consisting of 453 RCC tumors, panCREB1 expression was correlated to basic clinical patient data. PanCREB1 expression negatively and weakly correlated with pT stage, TNMV and TNML classification, and grading of the tumor, which is contradictory to observations in other tumors. Considering the high amount of locally restricted tumors in the consecutive cohort^45^, this finding should be interpreted with caution. There was also no significant association of panCREB1 expression to cancer-specific survival in our cohort. As shown in Fig. 1C, the CREB1 negative group was significantly smaller than CREB1 positive/high group, which could potentially cause a bias. In general, whether our data are in contrast to other solid tumors (e.g. esophageal squamous-cell carcinoma, glioma), in which CREB1 expression and activation was associated with higher TNM stage and grading^46,47^, needs to be discussed. However, in Hodgkin lymphoma, CREB1 was found to be tumor suppressive by regulating key aspects of cell proliferation. The authors showed that CREB1 depletion induces the expression of cell-cycle related genes and accelerates G1/S phase transition which in turn supports oncogenesis^48^.

In summary, an aberrant expression of the CREB1 protein in RCC was found. This deregulation might primary be a result of an altered post-transcriptional control. Indeed, we successfully identified four novel CREB1-regulating miRNAs by combining *in silico* analysis, an RNA affinity approach and molecular assays. These miRNAs were shown to be inversely expressed with CREB1 protein. Moreover, the analysis of an TMA consisting of 453 consecutive RCC tumors revealed a weak, but significant correlation of CREB1 with tumor stage, tumor grade TNM V and TNM L. To further validate the results of this study, the analyses of additional cohorts could be of interest. Subsequent studies should also focus on the phosphorylation state of CREB1 as this indicates the functional state of the protein.

## Material and Methods

### Cell lines, tissue culture and knock-down of CREB1 protein

In-house established ccRCC cell lines MZ2862RC, MZ2733RC, autologous normal kidney cell line MZ2733NN^49^ and HEK293T (purchased from the American Type Culture Collection) were maintained in Dulbecco’s Modified Eagle Medium (DMEM, Invitrogen) supplemented with 10% (v/v) fetal bovine serum (FCS) (PAA), 0,1 mM non-essential amino acids (Gibco), 2 mM L-glutamine (Lonza), and 1% penicillin/streptomycin (v/v; PAA) and incubated at 37 °C, 5% CO_2_.

Endoribonuclease-prepared siRNAs (esiRNA, Sigma Aldrich) targeting CREB1 were used to knock-down protein expression. 30 ng of esiCREB or esiCONTROL (specific for GFP) RNAs were used to transfect MZ2862RC cell line using Lipofectamine RNAi MAX (Invitrogen) according to the manufacturers’ protocol.

### Patients and tumor samples

This study was performed with the approval from the Clinical Research Ethics Committee of the Martin Luther University Halle-Wittenberg (“Ethik-Kommission”) and comprised 104 RCC patients who underwent surgery of the primary tumor at the Department of Urology, Martin Luther University Halle-Wittenberg. All experiments were performed in accordance with relevant guidelines and regulations. Signed informed consent was obtained from the patients prior to their participation in the study. Fresh frozen tumor samples were received immediately after surgical kidney resection. Patients’ characteristics were previously presented by Geissler and co-workers^50^.

### Protein isolation and Western blotting

Total protein was extracted using RIPA buffer (25 mM Tris-HCl pH 7.6, 150 mM NaCl, 1% NP-40, 1% sodium deoxycholate, 0.1% SDS) supplemented with protease and phosphatase inhibitors (ThermoFisher). Disruption and homogenization of tumor samples was achieved by the use of a TissueLyser II (Qiagen). Protein concentration was determined by Pierce™ BCA Protein Assay Kit (ThermoFisher).

For Western blot analysis, 25 µg of protein per sample was separated by SDS-PAGE and transferred onto a nitrocellulose membrane by semidry blot. For detection of proteins, following antibodies were used: CREB1 antibody 48H2 (CST), Argonaute-2 antibody C34C6 (CST), Maltose Binding Protein antibody ab9084 (Abcam), and β-actin antibody ab8227 (Abcam). As secondary antibody, a horseradish peroxidase conjugated goat α-mouse/rabbit antibody (CST) was used. Chemiluminescent blots were imaged by LAS-3000 Imaging System (Fuji). For densitometric analysis of western blot signal intensity ImageJ software^51^ was used.

### RNA isolation, cDNA synthesis and quantitative PCR

Total RNA isolation from formalin-fixed-paraffin-embedded (FFPE) tissue was performed using the AllPrep® DNA/RNA FFPE Kit from Qiagen (Hilden, Germany), according to the manufacturer’s protocol. Total RNA was isolated from cells using the NucleoSpin® miRNA kit (Macherey & Nagel) according to the manufacturers’ instructions followed by DNase I treatment (NEB). RNA quality and quantity were assessed by spectrophotometric analysis and 500 ng of total RNA was used for cDNA synthesis (RevertAid H Minus First Strand cDNA synthesis kit, Fermentas). For miRNA-specific cDNA synthesis a miRNA-specific stem-loop primer was used^52^, while reverse transcription of mRNAs required a random hexamer primer (Fermentas). Expression levels were analyzed by qRT-PCR using GoTaq® qPCR Master Mix (Promega). For all primer pairs, an annealing temperature of 61 °C was used. Relative changes of mRNA/miRNA amounts were determined by the ΔΔC_t_ method^53^ using GAPDH and U6 RNA for cell lines and miR-152^54^ for RNA derived from IHC samples for normalization. All used oligonucleotides are listed in Supplementary Table 2.

### RNA sequencing

Small RNA sequencing of RNA derived from the cell lines MZ2733RC and MZ2733NN was carried out at Novogene (Hong-Kong). In brief, sequencing libraries were generated using NEBNext® Multiplex Small RNA Library Prep Set for Illumina® (NEB, USA) following manufacturer’s recommendations. PCR products were purified on an 8% polyacrylamide gel and DNA fragments corresponding to ~140–160 bp were recovered. The library preparations were sequenced on an Illumina Hiseq 2500/2000 platform and 50 bp single-end reads were generated. Sequenced reads were mapped to miRBase20.0 provided reference^55^ and miRNA expression levels were estimated by TPM (transcript per million)^56^.

### miRNA enrichment assay (miTRAP)

For the identification of CREB1-specific miRNAs, the miTRAP method^24^ was employed. The detailed procedure was recently described^57^. Briefly, 3′-UTR of CREB1 (accession number NM_134442.5) was cloned as four parts (using the oligonucleotides listed in Table S1) upstream of two MS2 loops. T7 promoter based *in vitro* transcripts (T7 Ribomax, Promega) were purified using the MEGAclear Transcription Clean-Up Kit (Invitrogen). By application of 100 pmol of fusion protein consisting of the MS2 loop and maltose binding protein domains, *in vitro* transcribed RNAs (CREB1 3′-UTRs and control sequence encoding the two MS2 loops only) were immobilized on amylose resin (NEB). After washing and blocking steps, RNA was incubated with the cytoplasmic cell lysate of the ccRCC cell line MZ2733RC (CREB1 mRNA high/protein low) enabling the specific binding of miRNAs to the sequence. Extensive washing reduced unspecific binding and elution was carried out with 10 mM maltose solution followed by phenol/chloroform RNA extraction. RNA eluates were used for cDNA synthesis of candidate or already validated CREB1-specific miRNAs.

### Transfection, luciferase reporter gene assay and mutagenesis of miRNA binding sites

For miRNA mimic transfection, HEK293T cells were seeded at 1 × 10^5^ cells per well in 12-well plates and transfected with respective miRNA mimics or control (Sigma Aldrich) at a final amount of 10 pmol using Lipofectamine RNAi MAX (Invitrogen) according to the manufacturers’ protocol after 16 h.

For luciferase reporter assay, 1 × 10^4^ HEK293T cells per well were seeded in a 96-well plate. After 16 h, 10 ng of reporter plasmid (pmirGLO Dual-Luciferase miRNA Target Expression Vector, Promega) containing CREB1 3′-UTR sequence in combination with respective miRNA mimic or control (Sigma Aldrich) at a final concentration of 25 nM were transfected using Lipofectamine 2000 reagent (Invitrogen) according to the manufacturers’ protocol. Mutagenesis of the predicted miRNA binding site was enabled by NEBs Q5® Site-Directed Mutagenesis Kit using the oligonucleotides listed in Supplementary Table 1. Reporter assay was performed 48 h after transfection using the Dual-Glo® Luciferase substrate (Promega) according to the manufacturers’ protocol. Luminescence was detected by a Tecan Infinite 200 Pro plate reader device. Relative luciferase activity was determined by normalizing the *firefly* reporter to *renilla* luciferase activity.

### Tissue microarray and immunohistochemistry

Establishment of the consecutive RCC tissue microarray (TMA) consisting of 453 FFPE tumor samples of different subtypes, the associated patient and tumor characteristics and the procedure of immunohistochemical analysis have been recently described in detail^45^. Immunohistochemical staining (IHC) was performed by applying a 1:50 dilution of a rabbit monoclonal panCREB1-specific antibody (clone 48H2, #9197 Cell Signaling Technology) to 5 µM sections of the TMA. An HRP-linked polymer containing anti-rabbit (and anti-mouse) secondary antibody (Dako REAL™ EnVision™/HRP, Dako, Denmark) was employed and detection was visualized using 3,3′-diaminobenzidine chromogen substrate (Dako REAL™ DAB + Chromogen, Dako, Denmark). Staining evaluation and scoring was performed by two experienced observers (AH and CS). Staining intensity of each tissue core was recorded as 0 (absent), 1 (weak), 2 (moderate) or 3 (high). Simultaneously, the percentage of stained tumor cells was recorded as 0 (no stained tumor cells), 1 (1–9% stained tumor cells), 2 (10–50% stained tumor cells), 3 (51–80% stained tumor cells) or 4 (81–100% stained tumor cells). The intensity score of each sample was then multiplied by the percentage score it had achieved, i.e. values between 0 and 12 were possible. Finally, these results were grouped to negative (scores 0–2) and positive (scores 3–12).

### *In silico* analysis and statistics

For miRNA binding prediction different algorithms including Targetscan^25^, RNAhybrid^26^, miRanda^27^, and miRWalk2.0^28^ were used. Prism software (GraphPad) was used for calculating mean, standard deviation, t-test or Holm-Sidak method as well as displaying the results. For the two-sided t-test unequal variances have been selected. The data were significant with a p value <0.05.

IBM SPSS statistics Versions 21 and 24 were used for all the statistic tests on panCREB1 staining data. Two-sided exact Chi² tests (Pearson or Fisher’s, as appropriate) were calculated on panCREB1 staining distribution (negative vs. positive) among sex, age at diagnosis (≤65 years vs. >65 years), RCC subtypes (i.e. clear cell RCC, papillary RCC, chromophobe RCC and a heterogenous group of rare tumors), pT and WHO grade (2004). For calculation of correlation coefficients, Spearman Rho tests were performed on panCREB1 staining distributions (Score values 0 to 12), age at diagnosis (ungrouped and grouped (≤65 years vs. >65 years), T-stage, TNM L, TNM V and WHO grade (2004). Adjusted and non-adjusted correlations of panCREB1 protein expression measured by immunohistochemistry to overall survival were calculated by log rank tests. The Kaplan Meier Plot was drawn using R (R Foundation for statistical computing, version 3.6.1)^58^ and the package survminer^59^. P-Values <0.05 were regarded as significant. Details on the survival data of the cohort were published recently by Jasinski-Bergner *et al*.^45^.

## Supplementary information

Supplementary Information.

## Data Availability

Please contact author for data requests.
