# Supplementary material for: CREB1 is affected by the microRNAs miR-22-3p, miR-26a-5p, miR-27a-3p, and miR-221-3p and correlates with adverse clinicopathological features in renal cell carcinoma
Source: Sci Rep. 2020 Apr 16;10:6499. doi: 10.1038/s41598-020-63403-y (PMC7162877; doi:10.1038/s41598-020-63403-y)
Supplement: Supplementary file 1 — Supplementary Information. [file 41598_2020_63403_MOESM1_ESM.pdf]

CREB1 is affected by the microRNAs miR-22-3p, miR-26a-5p, miR-27a-3p, and miR-221-3p and correlates with adverse clinicopathological features in renal cell carcinoma

Michael Friedrich[1], Nadine Heimer[1], Christine Stoehr[2], André Steven[1], Sven Wach[3], Helge Taubert[3], Arndt Hartmann[2], Barbara Seliger[1*]

[1] Institute for Medical Immunology, Martin Luther University Halle-Wittenberg, 06112 Halle (Saale), Germany

[2] Institute of Pathology, University Erlangen-Nürnberg, 91054 Erlangen, Germany

[3] Institute of Urology, University Erlangen-Nürnberg, 91054 Erlangen, Germany

[*]Corresponding author:	Prof. Dr. Barbara Seliger<br>Martin Luther University Halle-Wittenberg<br>Institute for Medical Immunology<br>Magdeburger Str. 2<br>06110 Halle (Saale)<br>Germany
Telephone:	(+49) (345) 557 - 1357
Fax:	(+49) (345) 557 - 4055
E-mail:	barbara.seliger@uk-halle.de

Supplementary Data

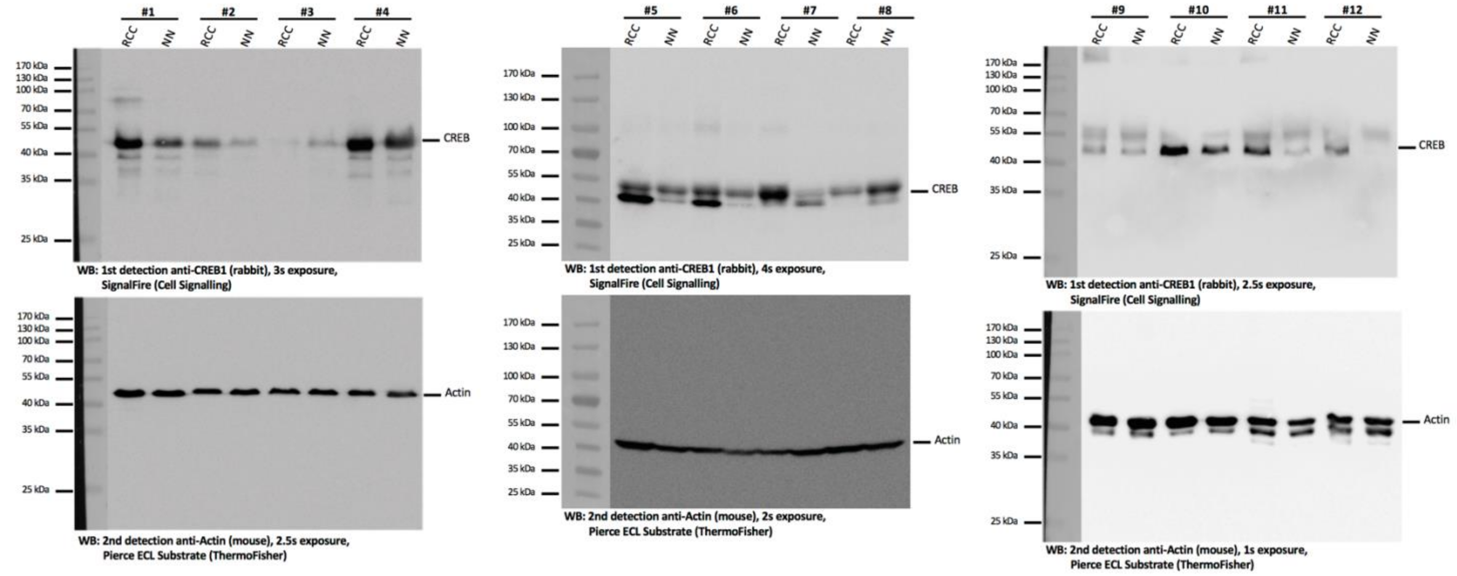

Supplementary Figure 1: Western blot-based detection of CREB1 in 12 pairs of ccRCC tumor samples (RCC) and matched adjacent normal tissue (NN). The same blot was re-probed for β-Actin serving as loading control.

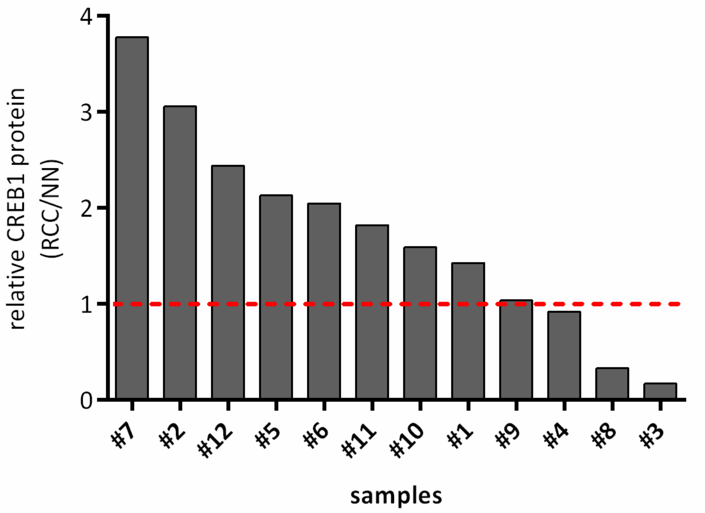

Supplementary Figure 2: Relative CREB1 protein quantification in RCC tumor samples and corresponding normal tissue based on the Western blot data shown in Figure 1A using β-Actin for normalization. Values >1 indicate higher CREB1 level in tumor samples, while values <1 indicate higher CREB1 level in corresponding normal tissue.

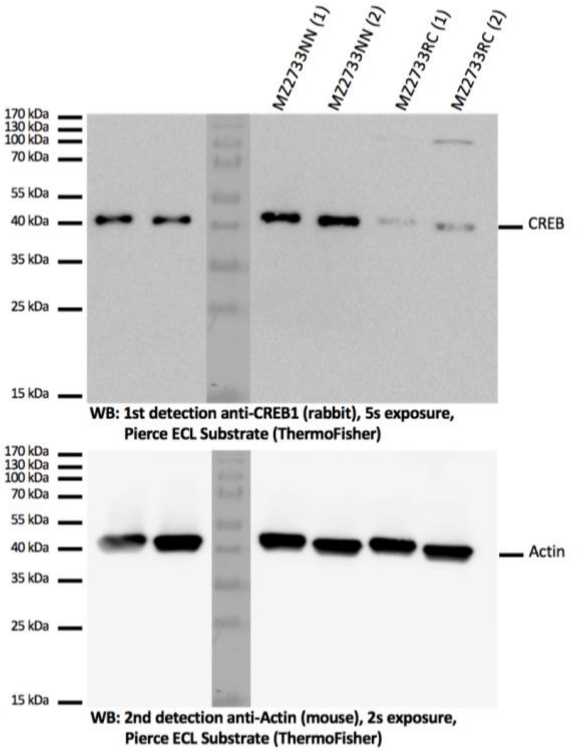

Supplementary Figure 3: Western blot-based detection of CREB1 protein in the ccRCC cell line MZ2733RC and the corresponding normal tissue cell line MZ2733NN. Number in parentheses indicates a technical replicate. Same blot was re-probed for β-Actin serving as loading control. Unlabeled lanes belong to an unrelated experiment.

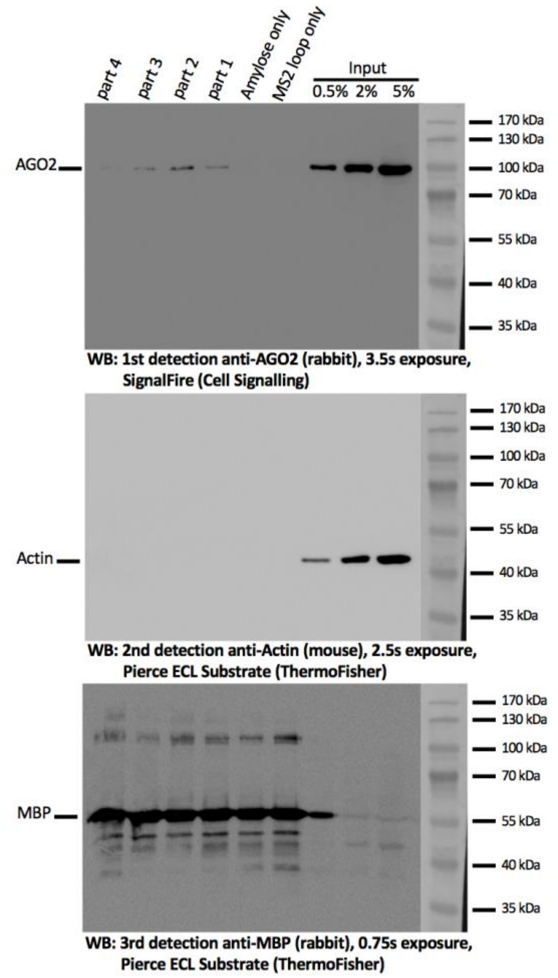

Supplementary Figure 4: Western blot-based detection of indicated proteins isolated from the miTRAP input sample (MZ2733RC) or co-precipitated with the used resin (amylose only), 2x MS2 loop RNA (MS2 loop only) or the different parts of the 3'-UTR of CREB1, respectively. Same blot was re-probed for β-Actin serving as negative control to exclude unspecific binding to the different RNAs. Detection of maltose binding protein (MBP) on the same blot ensures equal loading of the resin.

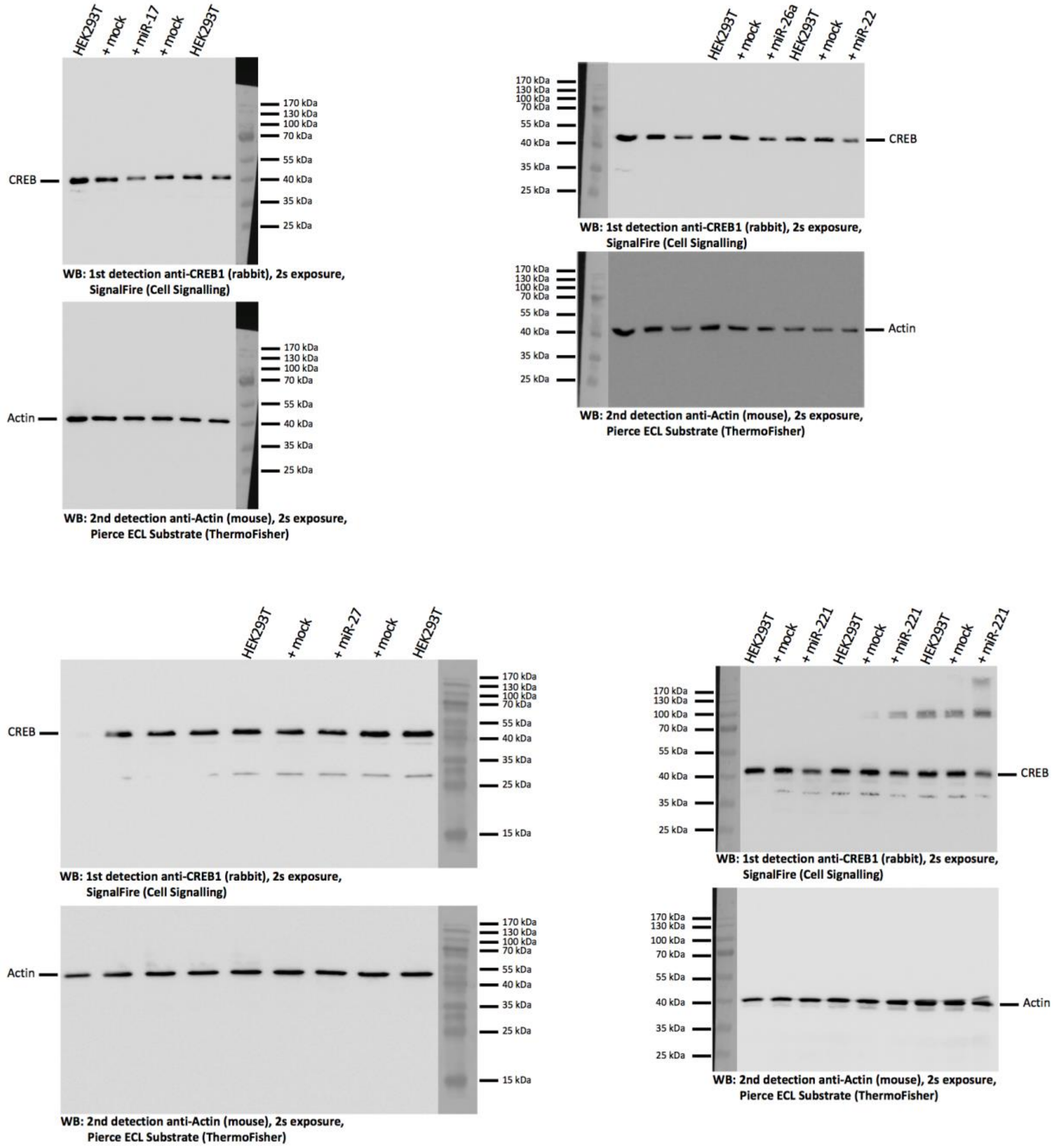

Supplementary Figure 5: Western blot-based detection of CREB1 after the miRNA mimic or mock transfection in HEK293T cells was performed as described in Materials and Methods. Same blot was re-probed for β-Actin serving as loading control. Unlabeled lanes belong to an unrelated experiment.

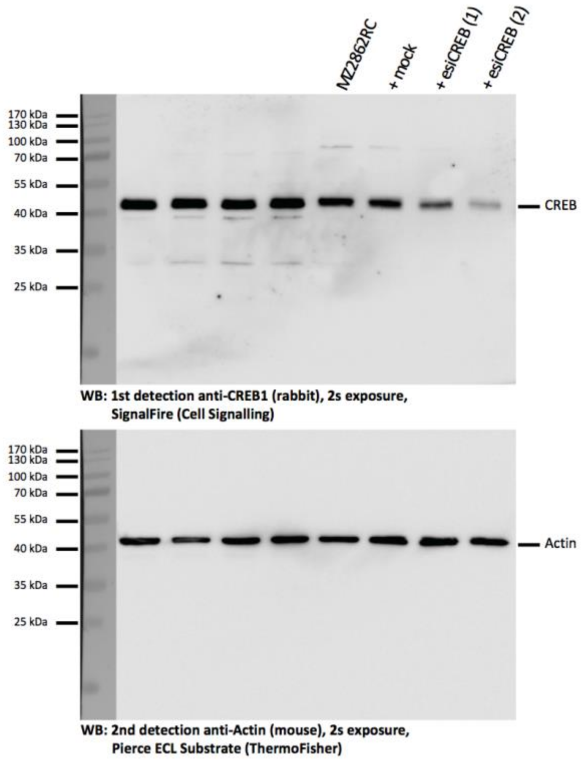

Supplementary Figure 6: Western blot-based detection of CREB1 after transfection of esiRNAs specific to CREB1 in MZ2862RC cell line. The number in parentheses indicates a technical replicate. Same blot was re-probed for β-Actin serving as loading control. Unlabeled lanes belong to an unrelated experiment.

Supplementary Table 1: Results of the Mantel Cox tests for correlation of panCREB1 to overall survival, adjusted for different co-variates.

co-variate		p-value	number of events	number of censored cases
age at diagnosis	< 65	0.155	24	133
	≥ 65	0.307	68	116
	pooled	0.106	92	249
sex	female	0.176	21	96
	male	0.413	71	152
	pooled	0.189	92	248
morphology	clear cell	0.234	69	197
	papillar	0.278	9	22
	chromophobe	0.747	4	17
	others	0.556	8	7
	pooled	0.100	90	243
tumor size pT	1	0.050	33	183
	2	0.099	12	22
	3	0.620	44	37
	4	n.a.	0	1
	pooled	n.a.	89	243
lymphovascular invasion (pL1)	0	0.579	59	164
	1	0.544	3	6
	pooled	0.495	62	170
vascular invasion (V1)	0	0.770	39	144
	1	0.456	16	21
	2	0.306	13	13
	pooled	0.739	68	178
WHO grade	1	0.652	4	35
	2	0.164	45	178
	3	0.337	43	35
	pooled	0.880	92	248

Supplementary Table 2: Oligonucleotides used in this study

name	sequence	application
3'UTR hCREB1 part1 fw	GGTACCTTTGGGATTTAAATTTTCACCTGTTAAGGT	amplification CREB 3'-UTR
3'UTR hCREB1 part1 rv	CTCGAGTTTGTGACTTTGCTTGATTTCTGTT	amplification CREB 3'-UTR
3'UTR hCREB1 part2 fw	GGTACCTTGTGTAACCGCATAGATATGTCA	amplification CREB 3'-UTR
3'UTR hCREB1 part2 rv	CTCGAGACAAGGTTTGAGCCCGTCTTT	amplification CREB 3'-UTR
3'UTR hCREB1 part3 fw	GGTACCGCACCTAAAAGCTAGCCTTAAAAAC	amplification CREB 3'-UTR
3'UTR hCREB1 part3 rv	CTCGAGCAAGGGGACCTGAGAAGGTA	amplification CREB 3'-UTR
3'UTR hCREB1 part4 fw	GGTACCCACGTGTTCACTGAAAGGACAA	amplification CREB 3'-UTR
3'UTR hCREB1 part4 rv	CTCGAGGTCTGTGCCACTTCTAATTTTAATTCCA	amplification CREB 3'-UTR
MF442_CREB Luc part1_fw	GCTAGCGGGATTTAAATTTTCACCTGTTAAGG	cloning CREB 3'-UTR in pmiR-GLO
MF443_CREB Luc part1_rv	GTCGACGACTTTGCTTGATTTCTGTTAAACC	cloning CREB 3'-UTR in pmiR-GLO
MF444_CREB Luc part2_fw	GCTAGCGTGTAACCGCATAGATATGTC	cloning CREB 3'-UTR in pmiR-GLO
MF445_CREB Luc part2_rv	GTCGACCTTTTAGGTGCTAAAATATTTTAATC	cloning CREB 3'-UTR in pmiR-GLO
MF446_CREB Luc part3_fw	GCTAGCGAAGGGAAAATAACTCAAGTGC	cloning CREB 3'-UTR in pmiR-GLO
MF447_CREB Luc part3_rv	GTCGACGGACCTGAGAAGGTATATAG	cloning CREB 3'-UTR in pmiR-GLO
MF522_mut.miR26_CREB	CAGGATAGGCTGTGACTTCAATCTAGATTTCTTTTAATATTTAAC	mutagenesis miRNA binding site
MF523_mut.miR26_CREB	GATTACTTGAAGGTTTAAACAAACGTATTTCTTC	mutagenesis miRNA binding site
MF524_mut.miR221_CREB	TGCTGGGTTTCTAAGCCTTTGTATGTGTC	mutagenesis miRNA binding site
MF525_mut.miR221_CREB	GACAATGTAGCTAACTTATCATAAGCAGATGAG	mutagenesis miRNA binding site
MF577_mut.miR27_CREB	TAAGTTCCGCGTGAATGTTCCAACACCTG	mutagenesis miRNA binding site
MF578_mut.miR27_CREB	GCCACTGTGAAGTTTAATGCAAAAGCACAAATG	mutagenesis miRNA binding site
MF579_mut.miR22_CREB	TGAAGAACTGTCTGTGGCAGCTATAACAG	mutagenesis miRNA binding site
MF580_mut.miR22_CREB	ACTGGCAGCTTCAAAGCATACAAACCAAATAC	mutagenesis miRNA binding site
MF8_Fw_GAPDH	AGGTCGGAGTCAACGGATTT	qRT-PCR
MF9_Rv_GAPDH	ATCTCGCTCCTGGAAGATGG	qRT-PCR
MF416_hCREB_fw	ACTGTAACGGTGCCAACTCC	qRT-PCR
MF417_hCREB_rv	GAATGGTAGTACCCGGCTGA	qRT-PCR
MF361_miR-9-5p_fw	CACGCATCTTTGGTTATCTAGC	qRT-PCR
MF360_miR-9-5p_st	TCGTATCCAGTGCAGGGTCCGAGGTATTCGCACTGGATACGACTCATAC	cDNA synthesis

name	sequence	application
miR-17-5p_qPCR	GCCCCAAAGTGCTTACAGTG	qRT-PCR
miR-17-5p_STL	GTCGTATCCAGTGCAGGGTCCGAGGTATTCGCACTGGATACGAC CTACCT	cDNA synthesis
miR-34b-5p_qPCR	GCCCTAGGCAGTGTCATTAGC	qRT-PCR
miR-34b-5p_STL	GTCGTATCCAGTGCAGGGTCCGAGGTATTCGCACTGGATACGAC CAATCA	cDNA synthesis
miR-181a-5p_qPCR	GCCCAACATTCAACGCTGTC	qRT-PCR
miR-181a-5p_STL	TCGTATCCAGTGCAGGGTCCGAGGTATTCGCACTGGATACGACA CTCAC	cDNA synthesis
miR-181b-5p_qPCR	GCCCAACATTCATTGCTGTC	qRT-PCR
miR-181b-5p_STL	GTCGTATCCAGTGCAGGGTCCGAGGTATTCGCACTGGATACGAC ACCCAC	cDNA synthesis
miR-200b-3p_qPCR	GCCCTAATACTGCCTGGTAA	qRT-PCR
miR-200b-3p_STL	GTCGTATCCAGTGCAGGGTCCGAGGTATTCGCACTGGATACGAC TCATCA	cDNA synthesis
miR-222-3p_qPCR	GCCCAGCTACATCTGGCTA	qRT-PCR
miR-222-3p_STL	GTCGTATCCAGTGCAGGGTCCGAGGTATTCGCACTGGATACGAC ACCCAG	cDNA synthesis
miR-22-3p_qPCR	GCCCAAGCTGCCAGTTGAAG	qRT-PCR
miR-22-3p_STL	GTCGTATCCAGTGCAGGGTCCGAGGTATTCGCACTGGATACGAC ACAGTT	cDNA synthesis
miR-26a-5p_qPCR	GCCCTTCAAGTAATCCAGGA	qRT-PCR
miR-26a-5p_STL	GTCGTATCCAGTGCAGGGTCCGAGGTATTCGCACTGGATACGAC AGCCTA	cDNA synthesis
miR-27a-3p_qPCR	CACGCATTCACAGTGGCTAAG	qRT-PCR
miR-27a-3p_STL	GTCGTATCCAGTGCAGGGTCCGAGGTATTCGCACTGGATACGAC GCGGAA	cDNA synthesis
miR-30a-5p_qPCR	GCCCTGTAAACATCCTCGAC	qRT-PCR
miR-30a-5p_STL	GTCGTATCCAGTGCAGGGTCCGAGGTATTCGCACTGGATACGAC CTTCCA	cDNA synthesis
miR-221-3p_qPCR	GCCCAGCTACATTGTCTGCTG	qRT-PCR
miR-221-3p_STL	GTCGTATCCAGTGCAGGGTCCGAGGTATTCGCACTGGATACGAC GAAACC	cDNA synthesis
miR-152-3p_qPCR	GCCCTCAGTGCATGACAGA	qRT-PCR
miR-152-3p_STL	GTCGTATCCAGTGCAGGGTCCGAGGTATTCGCACTGGATACGAC CCAAGT	cDNA synthesis
miRNA_rv	CCAGTGCAGGGTCCGAGGTA	qRT-PCR
